# Integrated Molecular and Bioinformatics Approaches for Disease-Related Genes in Plants

**DOI:** 10.3390/plants12132454

**Published:** 2023-06-26

**Authors:** Alpana Joshi, Hyung-Geun Song, Seo-Yeon Yang, Ji-Hoon Lee

**Affiliations:** 1 Department of Bioenvironmental Chemistry, College of Agriculture & Life Sciences, Jeonbuk National University, Jeonju 54896, Republic of Korea; joshi.alpana@gmail.com (A.J.); vkfkd1819@naver.com (H.-G.S.); 2 Department of Agriculture Technology & Agri-Informatics, Shobhit Institute of Engineering & Technology, Meerut 250110, India; 3 Department of Agricultural Chemistry, Jeonbuk National University, Jeonju 54896, Republic of Korea; dustj0501@gmail.com

**Keywords:** bioinformatics, physical mapping, plant pathogen, R-genes, CRISPR/Cas9, *NLRs*

## Abstract

Modern plant pathology relies on bioinformatics approaches to create novel plant disease diagnostic tools. In recent years, a significant amount of biological data has been generated due to rapid developments in genomics and molecular biology techniques. The progress in the sequencing of agriculturally important crops has made it possible to develop a better understanding of plant–pathogen interactions and plant resistance. The availability of host–pathogen genome data offers effective assistance in retrieving, annotating, analyzing, and identifying the functional aspects for characterization at the gene and genome levels. Physical mapping facilitates the identification and isolation of several candidate resistance (R) genes from diverse plant species. A large number of genetic variations, such as disease-causing mutations in the genome, have been identified and characterized using bioinformatics tools, and these desirable mutations were exploited to develop disease resistance. Moreover, crop genome editing tools, namely the CRISPR (clustered regulatory interspaced short palindromic repeats)/Cas9 (CRISPR-associated) system, offer novel and efficient strategies for developing durable resistance. This review paper describes some aspects concerning the databases, tools, and techniques used to characterize resistance (R) genes for plant disease management.

## 1. Background

Phytopathogens have greatly threatened livelihoods and societal growth because they affect quality crop production. Plant diseases caused by pathogenic bacteria, fungi, and viruses account for nearly 20–40% of losses in agricultural crop yields worldwide [1]. The molecular basis of the host–pathogen interaction is better understood due to the advancements in molecular and bioinformatics technologies. Whole-genome sequencing technology facilitates the sequencing of a large number of pathogens and plant species. Scientists are now able to organize and analyze enormous amounts of biological data using bioinformatics tools. Additionally, they can be used to identify and characterize disease-related genes and develop new diagnostic tools [2]. Plants have developed a multi-layered defense system against microbial diseases during evolution. The first level of protection is provided by the physical barriers imposed by the plant surface. The second layer is related to the detection of pathogen-associated molecular patterns (PAMP) that are anchored to the plasma membrane and activate the PAMP-triggered immunity (PTI) [3]. The third layer involves receptors encoded by resistance genes (R genes) that recognize the presence of pathogen-effector proteins and activate effector-triggered immunity (ETI) [4]. Plant disease resistance can be classified into two categories, namely qualitative resistance and quantitative resistance. Qualitative resistance is controlled by single resistance (R) genes, while the latter is controlled by multiple genes or quantitative trait loci (QTLs) [5]. Disease resistance mediated by resistance (R) proteins is associated with nucleotide binding (NB) and leucine-rich repeat (LRR) domains that are collectively known as NB-LRRs. The R genes are broadly categorized into eight classes based on their conserved protein structures. Resistance genes contain the CC-NBS-LRR (CNL) proteins that are characterized by a coiled-coil domain (CC), e.g., *RPM1* and *RPS2* genes of *Arabidopsis* and the *I2* resistance gene of *Solanum lycopersicum* (class I) [6,7]. The tobacco *N* gene and flax *L6* gene belong to class II (TIR-NBS-LRR (TNL), characterized by mammalian toll interleukin-1 receptor (TIR) and an NBS-LRR domain [8,9]. Some resistance genes belong to the RLK and RLP categories, such as *Cf-9*, *Cf-4*, and *Cf-2* for resistance to *Cladosporum fulvum*: (class III) [10,11,12]; *Xa21* for resistance to *Xanthomonas oryzae* (class IV) [13]; and *Ve1* and *Ve2* genes for resistance to *Verticillium* wilt (class V) [14]. Genome-wide studies of different classes of R genes have been reported in various plant species, including *Arabidopsis thaliana*, *Oryza sativa*, *Gossypium* sp., *Brassica napus*, *B. rapa*, *B. oleracea*, *Vitis vinifera*, *Triticum aestivum*, *Zea mays*, and *Hordeum vulgare* [13,15,16,17,18,19,20,21,22,23,24,25].

Exploiting genetic variation in natural populations is the key to plant improvements, whereas during co-evolution, pathogens adapted to their host and developed resistance against plant defense mechanisms. Alternatively, there is a need for new and advanced gene editing technologies to improve plant health, such as mega nucleases (MNs), zinc-finger nucleases (ZFNs), transcription activator-like effector nucleases (TALENs), and CRISPR (clustered regularly interspaced short palindromic repeats)/Cas (CRISPR-associated nucleases) [26]. The CRISPR/Cas system is a widely used genome-editing technology due to its easiness, low cost, high efficiency, and reproducibility. The CRISPR/Cas system is based on different strategies, including gene knock-out, knock-in, targeted mutagenesis, and modification of the amino acid sequence. For example, resistance against powdery mildew has been successfully developed in *T. aestivum*, *H. vulgare*, and *V. vinifera* by creating a knock-out mutant at the *MLO* locus (mildew resistance locus o) [27]. This system also develops resistance against multiple RNA viruses in *S*. *lycopersicum* and *Cucumis melo* by introducing INDELs affecting *eIF4Es* (eukaryotic translation initiation factor 4E proteins) [28,29]. Similarly, the CRISPR/Cas method has been successfully applied in developing resistance against bacterial, fungal, and viral diseases in diverse plant species, such as *A. thaliana*, *O. sativa*, *Glycine max*, *Malus domestica*, *Musa* species, *Nicotiana tabacum*, *Populus alba*, *S*. *lycopersicum*, *Solanum tuberosum*, *Sorghum bicolor*, *T. aestivum*, and *Z. mays* [26]. In this review, we focus on the characterization of R genes and the application of the CRISPR-Cas system to the development of resistance against specific pathogens.

## 2. Genome Databases of Plant Pathogens

Genome databases integrated with specific bioinformatics tools have been developed to study the associations between genetic diversity and disease (Table 1). They also provide information related to host–pathogen interactions. PhytoPath is a bioinformatics resource for genomic and phenotypic data of important plant pathogen species. The PhytoPath project utilizes the Ensembl genome portals to provide genomic information, including genome sequences, structural and functional annotation of protein-coding and non-protein coding genes, DNA and protein-based alignments, and phylogeny for genes [30]. The National Institute of Agrobiological Sciences (NIAS) Genebank is implementing the NIAS Genebank Project to preserve and document plant, microorganism, and animal genetic resources related to agriculture in Japan; however, it lacks a classification of plant gene functions [31]. The PathoPlant database has been developed to explain the molecular processes involved in signal transduction during plant pathogenesis and the interactions between plants and pathogens at the organism level [32]. The Pathogen-Host Interactions database (PHI-base) was established in the year 2005, and PHI-base entries include experimentally verified pathogenicity, virulence, and effector genes from fungal and bacterial pathogens of animal, plant, fungal, and other hosts [33]. The identification and analysis of host–pathogen interactions (HPI) are crucial to study infectious diseases. HPIDB 3.0 is a resource that helps to annotate, predict, and display host–pathogen interactions [34]. Viral infections often cause diseases by disturbing several cellular processes in the infected host. VirusMentha is a new resource for studying virus–virus and virus–host interactions based on integration techniques created for mentha, as well as the detailed curation protocols of the IMEx consortium [35]. An extensive database for predicting Penicillium-crop protein–protein interactions is PCPPI [36]. Currently, data can be amplified by extracting the information from microorganism genomes databases, but there is still a need for more extensive plant pathogen genome databases to understand the mechanism of disease resistance [37].

## 3. Identification and Isolation of Resistance (R) genes and Plant *NLRs*

Gene cloning is improving our understanding of the molecular mechanisms underlying plant–pathogen interactions. Map-based cloning or Positional cloning utilizes the knowledge of genetic map positions. It is the standard method to isolate genes when the phenotype and genomic locations are known. The first cloned R gene was *Hm1* from *Z. mays* against the HC toxin (the host-selective toxin pathogen) secreted by the fungus *Cochliobolus carbonum* [15]. Gene *Hm1* encodes a reductase enzyme that detoxifies the HC toxin and develops resistance in plants against *C*. *carbonum* followed by *Pto* (encoding a serine-threonine kinase) from *S. lycopersicum*, which confers resistance against *Pseudomonas syringae* pv*. tomato* [38]. Most isolated R genes encode cytoplasmic proteins consisting of a central nucleotide-binding site (NBS) domain and a C-terminal domain containing leucine-rich repeats (LRRs), including *Cf-9*, a predicted membrane protein with an extracellular LRR domain [10]. The *Cf-9* gene was isolated from *S. lycopersicon* through transposon tagging using the Maize Activator/Dissociation (Ac/Ds) system. Similarly, the *N* gene was isolated from tobacco (*N. tabacum*) via transposon tagging, and it conferred resistance to *Tobacco mosaic virus* (*TMV*) [8]. Furthermore, two genes (*RPS2* and *RPM1*) were isolated from *A. thaliana* conferring resistance against *P*. *syringae* using a map-based cloning approach [39,40], in addition to the *L6* gene in flax conferring against *Melampsora lini* using the Maize Activator/Dissociation (Ac/Ds) system [41]. Due to advancements in plant genomics and genetic engineering techniques, the positional cloning approach has made it easier to clone R genes from various crops or their wild relatives and transfer them into elite breeding lines or cultivars.

### 3.1. Plant NLRs

Nucleotide-binding site–leucine-rich repeats (NLRs) are encoded by hundreds of diverse genes per genome and can be divided into two major classes based on the presence of a distinct N-terminal domain: (i) CNL, containing a coiled-coil (CC) domain [6,7], and (ii) TNL, containing a Toll/interleukin-1 receptor (TIR) domain [8,9]. NLR proteins are abundant in plants, animals, fungi, and protists. Typically, several hundred *NLRs* are found in a plant genome [42], and the number, arrangement, and domain combinations of *NLRs* vary significantly in different plant species [43] (Table 2). For example, 3400 *NLRs* were identified in *T. aestivum* [44], 1000 *NLRs* in *M. domestica* [45], 535 *NLRs* in *O. sativa*, 245 *NLRs* in *S. bicolor*, 238 *NLRs* in *Brachypodium dystachyon* [46], 437 *NLRs* in *Gossypium hirsutum* [47], 459 *NLRs* in *V. vinifera*, 330 *NLRs* in *Populus trichocarpa* [48], 319 *NLRs* in *G. max* [49], 327 *NLRs* in *Manihot esculenta* [50], 571 *NLRs* in *M. truncatula*, 289 *NLRs* in *Cajanus cajan*, 337 *NLRs* in *Phaseolus vulgaris* [51], 151 in *Z. mays* [52], and 149 *NLRs* in *A. thaliana* [53]. Some plant species contain significantly low copy numbers of *NLRs*: for example, 54 *NLRs* in *Carica papaya* [52], 57 *NLRs* in *Cucumis sativus* [54], and 70, 55, and 55 *NLRs* in *C. sativus*, *C. melo*, and *Citrullus lanatus*, respectively [55]. Moreover, no correlation was observed between the total number of genes in the genome and genome size [46,56].

### 3.2. Resistance (R) Genes in Rice (O. sativa)

The rice crop is affected by several diseases, of which bacterial blight (BB) caused by *Xanthomonas oryzae* pv. *oryzae* is a serious disease that hinders the normal growth and production of rice. To date, 44 BB resistance genes have been discovered: 37 of which have been mapped and 15 have been cloned (*viz*., *Xa1*, *Xa2/Xa31*, *Xa3/Xa26*, *Xa4*, *Xa5*, *Xa7*, *Xa10*, *Xa13*, *Xa14*, *Xa21*, *Xa23*, *Xa25*, *Xa27*, *Xa41*, and *Xa45*) [13,25,58]. These isolated R genes can be classified into four groups based on their encoding proteins: (i) RLK (receptor-like kinase)—*Xa21* [13], *Xa3/Xa26* [59], and *Xa4* [60]; (ii) SWEET (sugar will eventually be exported transporter)—*Xa13* [61], *Xa25* [58], and *Xa41* [62]; (iii) executor genes—*Xa10* [63], *Xa23*, and *Xa27* [64]; and (iv) other types of genes—*Xa1* [65] and *Xa5* [66]. The other significant disease is rice blast, one of the most devastating diseases caused by the fungus *Magnaporthe oryzae*. More than 100 R genes have been identified, and 27 have been cloned *viz*., *Pib*, *Pb1*, *Pita*, *Pi9*, *Pi2*, *Pizt*, *Pid2*, *Pi33*, *Pii*, *Pi36*, *Pi37*, *Pikm*, *Pit*, *Pi5*, *Pid3*, *Pid3–A4*, *Pikh*, *Pish*, *Pik*, *Pikp*, *Pia*, *PiCO39*, *Pi25*, *Pi1*, *Pi21*, *Pi50*, and *Pi65R* [16,17,18,24,67,68,69]. *Pia* confers resistance to the blast fungus *M. oryzae* carrying the AVR-Pia, an avirulence gene, and a multifaceted genomics approach was employed to isolate the rice *Pia* gene [70,71,72,73]. Recently, new blast resistance genes were isolated, *Pi25* (resistance allele of *Pid3*) from a resistant cultivar Gumei2, the *Pi36* gene from the *indica* rice variety Kasalath, and *Pi-64(t)* and *Pi66(t)* from cultivar AS20-1. Moreover, the *Pi65(t)* gene was fine-mapped using a combination of bulk segregant analysis and next-generation sequencing, as well as *Pi-jnw1* from the *japonica* rice landrace Jiangnanwan [5,19,74] (Table 3).

### 3.3. Resistance (R) Genes in Wheat (T. aestivum)

Powdery mildew leaf rust (*Lr*)-resistance genes have been used successfully in different breeding programs to develop disease-resistant wheat cultivars. The first resistance genes, namely *Lr10*, *Lr21*, and *Lr1* against the fungal leaf rust disease caused by the pathogen *Puccinia triticina* were cloned in *T. aestivum* [75,76,77]. To date, more than 80 *Lr* genes have been characterized, and the majority of resistance genes (>50%) were derived from wild relatives of *T. aestivum*: (i) *Lr21*, *Lr22a*, and *Lr39* from *Aegilops tauschii*, (ii) *Lr24* from *Thinopyrum ponticum*, (iii) *Lr57* from *Ae. geniculate*, (iv) *Lr37*/*Yr17* from *Aegilops ventricosa*, (v) *Lr9* from *Aegilops umbellulata*, (vi) *Lr19* from *Thinopyrum elongatum* Zhuk., (vii) *Lr24* from *Agropyron elongatum*, (viii) *Lr26* from *Secale cereale* L, (ix) *Lr59* from *Aegilops peregrina*, (x) *Lr54* from *Aegilops kotschyi*, (xi) *Lr56* from *Aegilops sharonensis*, (xii) *Lr58* from *Aegilops triuncialis*, and (xiii) *Lr62* from *Aegilops neglecta* [78,79,80]. Similarly, more than 60 genes conferring resistance against stem rust (*Sr*) resistance have been identified in wild relatives of *T. aestivum viz*., *Sr5*, *Sr6*, *Sr7*, *Sr8*, *Sr9*, *Sr10*, *Sr13*, *Sr15*, *Sr16*, *Sr18*, *Sr19*, *Sr20*, *Sr21*, *Sr22*, *Sr23*, *Sr28*, *Sr29*, *Sr30*, *Sr33*, *Sr35*, *Sr41*, *Sr42*, *Sr45*, *Sr46*, *Sr48*, *Sr49*, *Sr50*, and *Sr60* [81,82,83,84,85,86,87,88,89]. Cereal cyst nematodes are serious pests affecting crop production. Resistance genes (*Cre*) were transferred into *T. aestivum* from its wild relatives to develop resistance against the root endoparasitic nematode *Heterodera avenae*, including *Cre1* and *Cre8* from *T. aestivum*; *Cre3* and *Cre4* from *A. tauschii*; *Cre2*, *Cre5*, and *Cre6* from *A. ventricosa*; *Cre7* from *A. triuncialis*; *CreR* from *S. cereale*; and *CreV* from *Dasypium villosum* [90].

Powdery mildew, caused by *Blumeria graminis f.* sp. *Tritici*, is a widespread disease in *T. aestivum* and responsible for severe yield loss. Resistance to powdery mildew has been associated with more than 140 genes in *T. aestivum* [91]. Map-based cloning and sequencing approaches have been employed to clone the resistance genes against powdery mildew, including *Pm2* [21], *Pm2a* [22], *Pm3* [92], *Pm3b* [93], *Pm3c* and *Pm3b* [94], *Pm5e* [24], *Pm8* [20], *Pm17* [95], *Pm21* [23], *Pm24* [96], *Pm41* [97], *Pm60* [98], *PmR1* [99], and *Pm2b* [100]. Mutant chromosome sequencing (MutChromSeq) is a method in which mutated chromosomes are sequenced and compared to the wild-type chromosomes to identify the novel target gene; for example, *Pm2a* located on chromosome 5DS was cloned using the MutChromSeq method. The *Pm3b* genes are located on chromosome 1AS and cloned using chromosome walking using available genetic resources. *Pm8* resistance genes have been introgressed from chromosome 1RS of *S. cereal* into *T. aestivum* using homology-based cloning. Similarly, *Yr10*, *Yr18*, *Yr36*, and *Yr46* genes have been isolated using a map-based cloning approach to develop genetic resistance against the fungal pathogen *Puccinia striiformis f.* sp*. tritici* [101,102]. Target-sequence Enrichment Sequencing (TEnSeq) pipelines were used to clone *Pm* genes, including *Pm1a* [103], *Pm2a* [21], and *Pm4b* [104]. Most of the cloned *Pm* genes contain an NLR, whereas resistance genes *Pm38* and *Pm46* encode an ATP-Binding Cassette (ABC) transporter [79] and a hexose transporter [78], respectively, which confer dual resistance to wheat leaf rust and stripe rust, in addition to resistance to powdery mildew (Table 4).

### 3.4. Resistance (R) Genes in Maize (Z. mays)

Fungal diseases are a major threat to maize production worldwide. Hm-l was the first gene cloned against the northern leaf spot fungus Cochliobolus carbonum [15]. Northern corn leaf blight (NCLB) is also one of the most devastating fungal diseases for maize caused by the fungal pathogen Setosphaeria turcica. The four resistance genes Ht1, Ht2, Ht3, and Htn1 against the fungal pathogen *S. turcica* have been identified and cloned using a map-based cloning approach. The dominant and race-specific Htn1 gene is effective against the most prevalent NCLB races. Htn1 encodes the wall-associated receptor-like kinase ZmWAK-RLK1, and the strength of the Htn1 resistance depends on environmental conditions and the maize genotype [105,106]. To date, only sixteen resistance genes (Hm1, Htn1, Ht2, Ht3, Rp1-D21, RppC, RabGD1α, ZmABP1, ZmAuxRP1, ZmCCoAOMT2, ZmCCT, ZmFBL41, ZmMM1, ZmREM1.3, ZmTrxh, ZmWAK) have been cloned from maize [107,108,109,110,111,112,113,114,115,116,117,118,119]. Southern corn rust (SCR) is the predominant disease in the USA, Canada, Brazil, and China, caused by Puccinia polysora. Although eleven maize dominant resistance genes (Rpp1, RPP6, RPP7, RPP8, Rpp9, Rpp10, and Rpp11) and eight major resistance QTLs (RppC, RppCML470, RppD, RppM, RppP25, RppQ, RppS, and RppS313) have been identified against the fungal pathogen P. polysora, only RppC was cloned [108]. Moreover, the RppK gene, which belongs to the CC-NB-LRR class, was cloned, via map-based cloning, and is involved in resistance against the same pathogen [110]. A major resistance quantitative trait locus, qRfg1, significantly enhances maize resistance to Gibberella stalk rot caused by Fusarium graminearum. A CCT domain-containing gene, ZmCCT, is the causal gene at the qRfg1 locus and was cloned using a map-based cloning approach [113]. ZmFBL41 was identified through a genome-wide association study in maize and confers resistance to banded leaf and sheath blight caused by the fungus Rhizoctonia solani [114]. Multiple disease resistance (MDR) is a valuable tool for developing durable resistance, and only one MDR gene (ZmMM1) has been cloned in maize. ZmMM1 confers resistance to northern leaf blight (NLB), gray leaf spot (GLS), and southern corn rust (SCR) [115].

Virus infections are also prevalent in maize-growing regions around the world. Maize rough dwarf disease (MRDD) is caused by various species of viruses belonging to the genus *Fijivirus*. The Rab GDP dissociation inhibitor alpha (RabGDIα) is the host susceptibility factor for rice black-streaked dwarf virus [111]. These resistance alleles are valuable to improve resistance to rough dwarf disease in maize and potentially develop resistance against rice *black-streaked dwarf virus* in other crops. *Sugarcane mosaic virus* (*SCMV*) is one of the severe viral diseases in maize. Two resistance loci, namely Scmv1 and Scmv2, conferring complete resistance against *SCMV* have been identified. Scmv1 encodes *ZmTrxh*, a molecular chaperone suppressing viral RNA accumulation in the cytoplasm without stimulating a salicylic acid- or jasmonic acid-mediated defense response [118,119] (Table 5).

### 3.5. Resistance (R) Genes in Arabidopsis (A. thaliana)

The cloning of resistance genes facilitates the development of resistant cultivars and develops an understanding of the evolutionary history of R genes. Most of the R genes identified in *Arabidopsis* belong to either the TIR-NBS-LRR or *LZ-NBS-LRR* subclass. In addition, receptor-like kinases (RLKs) are also involved in plant development and defense. The most well-known RLKs in *Arabidopsis* are the leucine-rich repeat receptor kinases flagellin-sensitive 2 (FLS2) and BAK1, which initiate the MAP kinase cascade upon flg22 recognition, leading to plant innate immunity [120,121]. The TIR-NBS-LRR subclass is defined by an N-terminal region that resembles the cytoplasmic domain of the Toll and interleukin1 transmembrane receptors (TIRs), e.g., *RPP1*, RPP4, and RPP5 confer resistance to *Peronospora parasitica* [122,123,124]. In contrast, the LZ-NBS-LRR subclass contains a leucine zipper–like motif (LZ) in place of the TIR domain, e.g., *RPS2*, *RPM1*, *RPP8*, and *RPP13* genes confer resistance to *P. syringae* [39,40,125,126]. Some R genes, *RPW7* and *RPW8*, encode proteins with motifs for a nucleotide-binding site (NBS), and an LRR confers resistance to the powdery mildew pathogens *Erysiphe cruciferarum* [127].

RPP4-mediated resistance has been associated with multiple defense-signaling components, including *EDS1* (enhanced disease susceptibility 1 [128], *NDR1* (non-race-specific disease resistance 1) [129], and *PBS1* [130], and the absence of functional alleles of either *EDS1* or *NDR1* leads to enhanced susceptibility to a diverse range of pathogens. In addition, EDS1 is required for RPS4-mediated disease resistance against *P. syringae* pv*. tomato* and does not specify resistance to *P. parasitica*, unlike other EDS1-dependent R genes [131]. The mapping and characterization of the *RCH2* locus identified the pair of neighboring genes, namely *RRS1* and *RPS4*, which confer dual resistance against fungal (*Colletotrichum higginsianum*) and bacterial (*Ralstonia solanacearum*) pathogens [132,133]. Similarly, map-based cloning has facilitated characterization of the *RFO* locus (RESISTANCE TO FUSARIUM OXYSPORUM (RFO), which is identical to *WAKL22* (WALL-ASSOCIATED KINASE-LIKE KINASE 22) in *Arabidopsis* [134]. *RPS5* belongs to the NBS-LRR subclass, and cloning *RPS5* genes has facilitated the characterization of two rps5 mutations. The rps5-1 mutation causes a glutamate-to-lysine substitution within the LRR region and affects the function of several R genes and confers resistance to both pathogens (*P*. *parasitica* and *P. syringae*) [135]. In *Arabidopsis*, members of both subclasses (TIR-NBS-LRR and *LZ-NBS-LRR)* confer resistance to the fungus *P*. *parasitica* and the bacterium *P*. *syringae*, whereas *RCY1*, belonging to CC-NB-LRR subclass, confers viral resistance. *Cucumber mosaic virus* (*CMV*) has the widest host range and causes catastrophic crop loss in many areas. *RCY1* is the dominant locus that confers resistance to the yellow strain of ecotype C24 in *Arabidopsis* [136] (Table 6).

### 3.6. Resistance (R) Genes in Tomato (S. lycopersicum)

The genome of tomato has been extensively explored to understand the structure and organization of resistance loci, and more than 100 loci have been identified [57]. The disease-resistance genes *Pto* [38], *Ptil* [137], and *Fen* [138] were discovered in *S. lycopersicum*, which confer resistance to bacterial speck caused by *P*. *syringae* pv*. tomato* (Pst). Another class of R genes, namely *Cf-2* and *Cf-9* from *Solanum pimpinellifolium* and *Cf-4* and *Cf-5* from *Solanum peruvianum*, have been identified and subsequently transferred into cultivated species to develop resistance against the leaf mold fungus *Cladosporium fulvum* [10,11,12,139]. Similarly, several other disease-resistance genes, including *Cf9* [10], *Cf5* [139], *Prf* ([140], *Sw5* [141], *I2* [142], *Mi1*-*2* [143], *Ve* [14], *Hero* [144], *Tm-2* [145], and *Bs4* [146], were cloned using molecular markers, chromosome walking, and linkage analysis. The *Sw-5* gene was introgressed from the wild species *S. peruvianum* to develop resistance against *TSWV* (*tomato spotted wilt virus*). Moreover, *Sw-5* was also found to be resistant to *Tomato chlorotic spot virus* (*TCSV*) and *Groundnut ring spot virus* (*GRSV*) [126]. Late blight caused by *Phytophthora infestans* is one of the most destructive diseases, and more than 60 resistance genes against *P. infestans* (*Rpi* genes) have been identified in *Solanum* sp. The *Ph*-*1* gene was the first reported *Rpi* gene (resistance against *P. infestans*), and after that, *Ph*-*2* and *Ph-2* genes have been identified in *S. pimpinellifolium* [132] and used to develop disease-resistant cultivars (Table 7).

## 4. NLR Annotation Tools

### 4.1. NLR-Parser

NLR-Parser is a tool to rapidly annotate the NLR (nucleotide-binding leucine-rich repeat) complement from sequenced plant genomes. It is a Java application used for the identification of NLR-like sequences. The pipeline was designed to use the MAST output from six-frame translated amino acid sequences and filters for predefined biologically curated motif compositions. Input reads can be derived from, for example, raw long-read sequencing data or contigs and scaffolds originating from plant genome projects. The output is a tab-separated file with information on the start and frame of the first NLR-specific motif, whether the identified sequence is a TNL or CNL, and whether it is potentially complete or fragmented. In addition, the output of the NB-ARC domain sequence can directly be used for phylogenetic analyses. NLR-parser can also discriminate pseudogenes by looking for the complete set of motifs defining an NLR protein. It uses motif alignment and a search tool (MAST) to search for 20 conserved motifs found in NLRs that use the highly-specific amino acid motif composition found in plant NLR gene products [148]. It can be downloaded from Git-Hub using the website (https://github.com/steuernb/NLR-Parser, accessed on 3 May 2023).

### 4.2. NLR-Annotator

NLR-Annotator is an extension of NLR-Parser to annotate NLR loci in genomic sequence data. Our pipeline dissects genomic sequences into overlapping fragments, and each fragment is translated in all six reading frames using NLR-Parser to preselect those fragments potentially harboring NLR loci. Using this approach, they could find putative candidate genes for NLR loci in stem rust, leaf rust, powdery mildew, and yellow rust resistance genes [44]. In 2018, NLR-Annotator, the improved version of NLR prediction, was released (https://github.com/steuernb/NLR-Annotator, accessed on 3 May 2023).

### 4.3. NLGenomeSweeper

Another pipeline to annotate functional NLR disease-resistance genes in genome assemblies is NLGenomeSweeper. It is a pipeline that searches a genome for NBS-LRR (NLR) disease-resistance genes based on the presence of the NB-ARC domain. This procedure can be used with a customized NB-ARC HMM consensus protein sequence(s) created for a species of interest for each type of NBS-LRR (TNLs, CNLs, and NLs) and merge them into a single fasta file for use. This pipeline shows high specificity for complete genes and structurally complete pseudogenes. This pipeline identified 152 potential NBS-LRR proteins; 140 of these matched the manually annotated *Arabidopsis* NLR set, which contains 146 genes (96% sensitivity) [149].

### 4.4. DRAGO2

Disease Resistance Analysis and Gene Orthology (DRAGO 2) is the second version of a pipeline to annotate resistance genes. It is an extensive, freely accessible, and user-friendly online platform for analyzing and predicting plant disease-resistance genes. The input of DRAGO 2 can be either DNA or protein sequences in FASTA format. DRAGO2 is available in PRGdb (http://prgdb.org, accessed on 3 May 2023). The core of the DRAGO2 pipeline is a Perl script that predicts putative pathogen receptor genes (PRGs) and LRR, kinase, NBS, and TIR domains. It can also detect CC and TM domains using COILS 2.2 and TMHMM 2.0c programs. More than 1700 possible PRGs were predicted using the DRAGO2 tools, which have the highest sensitivity compared to other tools [150].

### 4.5. NLRtracker

NLRtracker has been designed to overcome the limitation associated with the existing NLR tools. NLRtracker uses InterProScan and the predefined NLR motifs to annotate all sequences in a given proteome or transcriptome and then extracts and annotates *NLRs* based on the core NLR sequence features (late blight R1, TIR, RPW8, CC, NB-ARC, LRR, and integrated domains) found in the RefPlantNLR dataset. Additionally, NLRtracker extracts the NB-ARC domain for a comparative phylogenetic analysis [151].

## 5. CRISPR Gene Editing for the Generation of Disease Resistance

The CRISPR (clustered regulatory interspaced short palindromic repeats)/Cas9 (CRISPR-associated) system has surpassed alternative genome editing methods due to its simplicity, flexibility, better success rate, and cost-effectiveness. The CRISPR/Cas9 system can efficiently introduce mutations, including INDELs (insertion mutations and deletion) and base substitutions in the target site. One significant advantage of using the CRISPR/Cas9 system is the ability to edit multiple target genes simultaneously [152]. Several efficient plant genome editing web-based tools are available for designing sgRNAs and analyzing post-genome editing data [153] (Table 8). CRISPR/Cas systems have been divided into six types based on their signature Cas genes. Class 1 CRISPR/Cas systems (types I, III, and IV) employ multi-Cas protein complexes for interference, whereas class 2 systems (types II, V, and VI) accomplish interference with single effector proteins in complex with CRISPR RNAs (crRNAs) [154]. This system has been successfully applied to various plant species, such as *A. thaliana*, *O. sativa*, *N. tabacum*, *S. bicolor*, *T. aestivum*, *Z. mays*, *G. max*, *S. lycopersicum*, *S. tuberosum*, *P. alba*, *M. domestica*, and *Musa* species, to combat viral infection and fungal and bacterial diseases [26,155]. There are several strategies for developing plant disease resistance via the CRISPR/Cas system [156]: (i) knock-out of susceptibility genes of disease (e.g., *MLO*; a mildew resistance locus O) [27], (ii) deletion or modification of cis-elements in promoters [157], (iii) modification of the amino acid sequence of surface receptor proteins to suppress secreted pathogen effectors [153], (iv) knockdown of negative regulators of plant immunity [158], and (v) modification of central regulators of the defense response [159].

The CRISPR/Cas9 system has facilitated efficient and precise targeted mutagenesis in plants to enhance resistance to fungal diseases. Mildew resistance locus O (MLO) is the most widely studied gene for resistance to fungal diseases. Wild-type alleles of *MILDEW RESISTANT LOCUS O* (*Mlo*) are conserved throughout monocots and dicots, conferring susceptibility to the powdery mildew fungi *Oidium neolycopersici*. The generation of a resistant variety using CRISPR/Cas9 technology against the powdery mildew pathogen was reported in various crops: *H. vulgare*, *A. thaliana*, *S. lycopersicum*, *Pisum sativum*, *Fragaria vesca*, *Capsicum annuum*, *T*. *aestivum*, *C. sativus*, *Rosa hybrid*, *N. tabacum*, *C. melo*, *V. vinifera*, and *M. domestica* [27]. *SlMlo1* is a major gene responsible for powdery mildew disease in *S. lycopersicum*, among 16 *MLO* genes studied so far. CRISPR/Cas9 technology has been employed to knock out SlMlo1 in developing resistance against the powdery mildew fungus *O. neolycopersici* without affecting the phenotype [27]. Similarly, a CRISPR/Cas9 system was used to mutate the susceptibility gene of *Powdery Mildew Resistance 4* (*PMR4*), resulting in resistance to *O. neolycopersici* in *S. lycopersicum*. Additionally, both TALENs and CRISPR tools have been used to introduce mutations in one (*TaMLO-A1*) of the three *MLO* homoalleles, which resulted in improved resistance against *B. graminis* f. sp. *tritici* infection in *T. aestivum* [160,161]. In a similar study, a CRISPR-mediated *MLO* mutation resulted in the development of resistance to powdery mildew in *H. vulgare* (*B. graminis f.* sp. *hordei*), but at the same time, it increased susceptibility to the blast fungus *M. grisea* (*M. oryzae*) in *O. sativa* [162]. The CRISPR/Cas9-mediated editing of two susceptible genes, *MLO-6* and *DMR*, resulted in increased resistance against the powdery mildew fungus *Erysiphe necator* and downy mildew fungus *Plasmopara viticola* in *V. vinifera* [163]. Another study in *V. vinifera* demonstrated that loss of the *VvMLO7* gene increased resistance against *E. necator* through gene knock-down [164,165]. The CRISPR/Cas9-mediated knock-out of two genes, *Solyc08g075770* and *SlymiR482e-3p*, in the different studies, resulted in resistance against the pathogen that causes Fusarium wilt in *S. lycopersicon* [166,167]. Similarly, a mutation in the *Clpsk1* gene enhanced resistance against *F. oxysporum* in *C. lanatus* [168].

*EDR1* (*enhanced disease resistance*) is highly conserved across plant species and negatively affects plant immunity. In *Arabidopsis*, *EDR1* was reported to be a negative regulator of powdery mildew resistance, and this regulation was mediated by suppressing salicylic acid and enhancing abscisic acid signaling. Three homologs of the *TaERD1* gene were mutated using CRISPR/Cas9, and the resultant *Taedr1*-mutant plants showed a significant reduction in blast lesions and resistance to powdery mildew in *T. aestivum* [169]. It was reported that the expression of *EDR1* was induced by jasmonic acid (JA), salicylic acid, ethylene, and abscisic acid [170]. Moreover, both jasmonic and salicylic acid accumulation is associated with enhanced resistance against *X. oryzae* pv. *oryzae* (*Xoo*) in *O. sativa*. OsEDR1-knock-out plants demonstrated enhanced resistance against the bacterial blight-causing pathogen *Xoo* [171]. *DMR6* (*downy mildew resistance 6*) has been identified as a susceptibility gene in *S. tuberosum* [172] and *Arabidopsis* [173]. Two DMR genes (*StDMR6-1* and *StDMR6-2*) were edited simultaneously in *S. tuberosum* resulting in enhanced resistance against the late blight fungus *P. infestans* [174].

Rice blast is one of the most devastating diseases that affect rice production worldwide. *Ethylene-responsive factors* (*ERFs*) of the *APETELA2/ERF* (*AP2/ERF*) superfamily play crucial roles in adaptation to various biotic stress. Rice blast resistance to the fungus *M. oryzae* was enhanced mediated through the CRISPR/Cas9-mediated mutation of *ERF922* gene [175]. Knock-down of the AP2/ERF transcription factor reduced abscisic acid accumulation and increased resistance against *M. oryzae* [176]. Similarly, the CRISPR/Cas9-mediated knock-out of AtERF019 in *A. thaliana* enhanced resistance to *Phytophthora parasitica* by suppressing PAMP-triggered immunity [177]. The overexpression of defense genes is one of the key biotechnological tools exploited to develop resistance against plant pathogens. In *Theobroma cacao*, overexpression of the *TcNPR1* (*Non-expressor of Pathogenesis-Related 1*) gene reduced infection caused by *Phytophthora* spp. in leaf tissue [158].

*Microrchidia* (*MORC*) proteins are important nuclear regulators in prokaryotes and eukaryotes, involved in transcriptional gene silencing and the maintenance of genome stability [178]. In *Arabidopsis*, the role of *MORC1* was discovered in plant immunity against *turnip crinkle virus* (*TCV*). Moreover, the role of *AtMORC1*, *AtMORC2*, and *AtMORC6* are reported in multiple layers of defense responses against *P*. *syringae* and *Hyaloperonospora arabidopsidis* [49,179]. The CRISPR-Cas9 system from *Streptococcus pyogenes* (CRISPR/*Sp*Cas9) was used to introduce a mutation at *HvMORC1* and *HvMORC6a* genes in *H. vulgare*. Similarly, *MORCs* have also been studied in *S. tuberosum* (*StMORC1*), *S. lycopersicum* (*SlMORC1*), and *Nicotiana benthamiana* (*NbMORC1*) [180,181]. *WRKYs* (*WRKY* transcription factors) have been identified in different plants in plant immune responses. Mutant analyses in *Arabidopsis* have revealed direct links between specific *WRKY* proteins (*WRKY8*, *WRKY11*, *WRKY33*, *WRKY38*, *WRKY53*, *WRKY62*, and *WRKY70*) and defense responses against *P*. *syringae*. Coronatine (COR) is the phytotoxic compound produced by the pathogen *P*. *syringae* pv tomato DC3000 (Pto3000), causing bacterial speck disease in *S. lycopersicon*. The CRISPR/Cas9-mediated mutation of the *S1JAZ2* gene resulted in resistance to bacterial speck disease infestation in *S. lycopersicum* [182]. The role of the *WRKY70* gene in the disease response to the fungus *Sclerotinia sclerotiorum* in *B. napus* was also documented in the literature [159]. In a similar study, the CRISPR/Cas9-mediated targeted mutagenesis of *VvWRKY52* produced mutant lines in *V. vinifera* and the knock-out of *WRKY52* enhanced resistance to *Botrytis cinerea*, causing gray mold disease [165].

Many viruses infecting economically important crops belong to the category of RNA viruses. CRISPR/Cas technology has been applied successfully to develop resistant plants against RNA viruses. Rice tungro disease is a severe problem caused by an interaction between *rice tungro spherical virus* and *rice tungro bacilliform virus.* In plants, *eIF4E* and *eIF(iso)4E* assist in recruiting ribosomes to the 5′ UTRs of mRNAs, which is eventually required to translate viral proteins. The copy numbers of the *eIF4E* and *eIF(iso)4E* genes vary from species to species [183]. A CRISPR/Cas9-mediated mutation in *eIF4G* provided resistance to *rice tungro streak spherical virus* in a susceptible variety (IR64) of *O. sativa* [184]. Mutation of the recessive *eIF4E* gene enhanced resistance against *turnip mosaic virus* in *Arabidopsis* and *cucumber vein yellowing virus* in cucumber [185,186]. Similarly, RNA virus resistance has been demonstrated by silencing the *eIF4E* gene in *S. lycopersicum* and *C. melo* [28,29]. A recent discovery of *FnCas9* (Cas endonucleases) from *Francisella novicida* may be used as a new tool for attacking the genome of plant RNA viruses. *FnCas9* was used to develop resistance against *Cucumber mosaic virus* (*CMV*) and *Tobacco mosaic virus* (*TMV*) in *N. benthamiana* and *Arabidopsis* plants, respectively [187]. Characterization of the functionality of *Cas13a* of *Leptotrichia shahii* (LshCas13a) demonstrated that the single effector *Cas13a* protein was a programmable RNA-guided single-stranded RNA (ssRNA) ribonuclease that provided immunity against bacteriophages of the bacteria *Escherichia coli* [188]. The LshCas13a system was used for developing resistance to *Southern rice black-streaked dwarf virus* (*SRBSDV*) and *Rice stripe mosaic virus* (*RSMV*) in *O. sativa* [189].

*O. sativa* is extensively used for genome editing studies against bacterial disease resistance. Rice bacterial blight is one of the invasive diseases caused by bacterial *X. oryzae* pv*. oryzae* (*Xoo*) [190]. *X. oryzae* secretes transcription-activator-like effectors (TALes) that bind specific promoter sequences and induce sucrose transporter genes (*SWEET11*, *SWEET13*, and *SWEET14*). The expression of sucrose transporter genes is required for disease susceptibility and mutations in effector binding element (EBE) regions in promoters of *SWEET11*, *SWEET13*, and *SWEET14* genes [157]. The CRISPR/Cas9-mediated knockout of the *Os8N3* gene resulted in enhanced resistance to most *Xoo* and bacterial blight [191]. Similarly, induced mutations in *O. sativa* into the coding regions of *TMS5* (thermosensitive male sterile), *Pi21* (proline-rich protein), and *Xa13* (bacterial blight resistance) genes via CRISPR/Cas9 improved resistance against rice blast and bacterial blight [192]. The genus *Xanthomonas* is one of the significant genera affecting various horticultural crops. Citrus canker is one of the major diseases of citrus caused by the bacterium *Xanthomonas citri* ssp*. citri* (*Xcc*). *Lateral Organ Boundaries 1* (*CsLOB1*) is a transcription factor that assists in the proliferation of *X. citri* spp. *citri* (*Xcc*). Effector binding element (EBE) regions present in the *CsLOB1* promoter are recognized by the *Xcc* effector (*PthA4*), and expression of the *CsLOB1* gene facilitates canker development in *Citrus* sp. CRISPR/Cas9-mediated editing of EBEs in the *CsLOB1* promoter and coding region of the *CsLOB1* gene provides resistance to citrus canker in *C. sinensis* and *C. paradise* [193]. Similarly, another transcription factor, *WRKY22***,** was mutated through CRISPR/Cas9 technology and resulted in resistance to citrus canker in *C. sinensis* [194]. Fire blight is another devastating disease caused by *Erwinia amylovora* in *M. domestica.* The CRISPR/Cas9-mediated mutation of disease-specific interacting protein (*DIPM-1*, *DIPM-2*, and *DIPM-4*) genes provides resistance to the golden delicious variety of *M. domestica* against fire bight disease [195]. The application of the CRISPR/Cas system for disease resistance development by either targeting the pathogen genome or host genes to interfere with susceptibility has become more effective due to its simple operation, good knockout effect, low cytotoxicity, high specificity, and universal applicability. The CRISPR system has attracted more and more attention because CRISPR/Cas-induced mutations create pathogen-resistant genotypes when resistance resources in natural populations or wild relatives are limited. CRISPR/Cas also offers the opportunity to develop designer plants with multiple valuable attributes and resistance against biotic and abiotic stress. Thus, this technology should be explored and improved for creating novel disease-resistance genes/genotypes, which ultimately need reduced pesticide applications. These developments in genome editing will undoubtedly be advantageous for environmentally sustainable agriculture.

Intracellular nucleotide-binding leucine-rich repeat (NLR) receptors recognize pathogen effectors and initiate the immune response. The mechanisms of plant NLR activation remain unresolved, whereas animal NLRs undergo oligomerization upon binding to their effectors to activate downstream signaling. Our understanding of the plant NLR activation process has greatly increased due to the available structural data of CNL and TNL resistosomes. The composition and three-dimensional CNL structures of an *Arabidopsis ZAR1* (*HopZ-activated resistance*) using cryo-EM microscopy structures illustrate differences between inactive and intermediate states of *ZAR1* [196]. Similar studies uncovered the CNL structure of wheat *Sr35* and found its resemblance to the ZAR1 resistosome structure of *Arabidopsis* [83,197]. In addition, the cryo-EM structures of TNL resistosomes from *RPP1* (recognition of *Peronospora parasitica 1*) and *ROQ1* (recognition of *Xanthomonas outer protein Q 1*) from *A. thaliana* and *N. benthamiana*, respectively, were determined using cryo-EM microscopy [198,199]. Recent advancements in computational methods, such as AlphaFold, have been used to predict the three-dimensional structure of the protein *AVRamr1* (recognition of *P. infestans* effector) [200]. This structural framework moves us closer to developing novel immune receptors with modified recognition specificities and more effective plant disease-resistance proteins. Modern technology recognizes potential target regions of NLRs and the conserved resistosome structure, highlighting the future possibility of crop improvement through structure-guided NLR engineering. However, some questions are yet to be answered, such as whether all CNL and TNL immune receptors exhibit resistosome properties or if NLR activation requires the resistosome, as well as the possibility of monitoring resistosome formation using engineered NLR chimera.

## 6. Conclusions

NLRs play a crucial role in plant immunity by activating the strong resistance response leading to plant disease resistance. NLRs have a central nucleotide-binding (NB) domain which acts as an on/off activation switch, followed by a leucine-rich repeat (LRR) domain. The structure diversity, abundance, and chromosomal distribution of NLRs are fundamental for understanding disease resistance. The availability of high-throughput sequencing technology allows for the identification and cloning of several candidate resistance genes in different plant species. Gene editing technologies create a novel variation at the gene and genome levels. However, pathogens can eventually overcome disease resistance based on single-base editing due to their rapid evolution and genetic diversity of bacterial and fungal populations. The advanced variants of genome-editing tools, such as CRISPR/Cas, have brought many insights into the molecular mechanisms of site-specific mutagenesis. Moreover, durable resistance can be produced by pyramiding numerous genes and/or altering the plant and pathogen genomes using CRISPR/Cas9 technology. Protein engineering has redefined our ability to develop new or improved molecular recognition capabilities of NLRs, and engineered intracellular immune receptors can potentially improve disease resistance. The research on NLR proteins has been limited due to the unavailability of adequate three-dimensional structures of individual domains and homology models. However, in recent years, a significant advance in cryo–electron microscopy resolved the full-protein cryo-EM structure of NLR complexes, providing comprehensive insights into the complex biological mechanisms and functional complexity of NLRs. Moreover, modern computational technology, such as Alphafold, ca predict the three-dimensional structures of proteins with higher accuracy. These cutting-edge technologies may generate designer NLR receptors to confer broad-spectrum resistance in crop plants. Furthermore, more comprehensive tools are required for understanding accurate protein structures, ligand binding, and host–pathogen interactions. Overall, integrated computational and molecular biology tools provide a practical approach for efficiently breeding multiline cultivars and a strategy for generating designer crops with broader resistance and high yields.

## Figures and Tables

**Table 1:** Databases related to important plant pathogen species.

Database	Data Sources	Main Pathogens	Analysis Tool	URL
PhytoPath [30]	Ensembl Genomes, PHI-base	Bacteria, fungi, and protists	Ensembl data visualization	http://www.phytopathdb.org/ (accessed on 2 May 2023)
NIASGBdb [31]	Experimental data and published literature	Bacteria, fungi, and viruses	--	http://www.gene.affrc.go.jp/databases_en.php (accessed on 2 May 2023)
PathoPlant [32]	GenBank, SWISS-PROT, TRANSFAC, PubMed and published literature	Bacteria, fungi, viruses, and nematodes	In silico expression analysis	http://www.pathoplant.de/ (accessed on 2 May 2023)
PHI-base [33]	NCBI, EMBL, and Web of Science	Bacteria, fungi, and protists	PHI-BLAST	http://www.phi-base.org (accessed on 2 May 2023)
HPIDB [34]	IntAct, MINT, BioGRID, HPIDB, BIND, and VirHostNet	Bacteria, fungi, and viruses	BLAST, visualization of interaction network	https://hpidb.igbb.msstate.edu/ (accessed on 2 May 2023)
VirusMentha [35]	MatrixDB, BioGRID, MINT, IntAct, and DIP	Virus	Visualization of interaction network	http://virusmentha.uniroma2.it/ (accessed on 2 May 2023)
PCPPI [36,37]	By predicting	Fungi	BLAST, visualization of interaction network	http://pcppi.atcgn.com/blast.html (accessed on 2 May 2023)

BIND—The Biomolecular Interaction Network Database BioGRID (Biological General Repository for Interaction Datasets, DIP—Database of interacting proteins, EMBL—European Molecular Biology Laboratory, MINT—the Molecular INTeraction database, NCBI—National Center for Biotechnology Information, PHI-base—Pathogen-Host Interactions database, TRANSFAC—TRANScription FACtor database, VirHostNet*—*Virus–Host Network.

**Table 2:** Distribution of NLR gene family in plant species.

Species	CC-NBS	CC-NBS-LRR	NBS-LRR	TIR-NBS	TIR-NBS-LRR	References
*Oryza sativa*	77	156	70	-	-	[46]
*Hordeum vulgare*	60	198	84	-	-	[44]
*Triticum urartu*	78	275	107	-	-	[44]
*Aegilops tauschii*	70	298	113	-	-	[44]
*Triticum aestivum*	493	1181	367	-	-	[44]
*Zea mays*	93	151	-	-	-	[51]
*Brachypodium distachyon*	53	201	60	-	-	[46]
*Vitis vinifera*	26	200	12	14	90	[48]
*Populus trichocarpa*	14	119	-	10	73	[48]
*Manihot esculenta*	11	117	43	5	29	[50]
*Medicago truncatula*	16	94	139	49	121	[51]
*Cajanus cajan*	7	63	68	6	78	[51]
*Phaseolus vulgaris*	9	128	96	13	76	[51]
*Glycine max*	8	109	137	24	124	[51]
*Arabidopsis thaliana*	5	51	3	21	93	[53]
*Solanum lycopersicon*	35	123	48	9	21	[57]

**Table 3:** Resistance genes, their donor parents, chromosomes location, and cloning techniques in *O. sativa*.

Source	R-Gene	Disease	Pathogen	Gene Product	Chromosome	Cloning Technique	References
*O. sativa*	*Xa1*	Bacterial blight	*X. oryzae*	NBS-LRR	4	Map-based cloning	[65]
*O. sativa*	*Xa5*	Bacterial blight	*X. oryzae*	NBS-LRR	5	Map-based cloning	[66]
*O. sativa*	*Xa10*	Bacterial blight	*X. oryzae*	Transcription activator-like (TAL) effector	11	Map-based cloning	[63]
*O. sativa*	*Xa13*	Bacterial blight	*X. oryzae*	--	8	Map-based cloning	[61]
*O. sativa*	*Xa21*	Bacterial blight	*X. oryzae*	Receptor kinase-like protein	11	Map-based cloning	[13]
*O. sativa*	*Xa25*	Bacterial blight	*X. oryzae*	Transmembrane domain	12	Map based cloning	[58]
*O. sativa*	*Xa3/Xa26*	Bacterial blight	*X. oryzae*	eLRR-TM-kinase or LRR receptor-kinase proteins	11	Map-based cloning	[59]
*O. minuta*	*Xa27*	Bacterial blight	*X. oryzae*	Receptor kinase-like protein	6	Map-based cloning	[64]
*O. sativa*	*Pi36*	Bacterial blight	*M. oryzae*	CC-NBS-LRR	8	Map-based cloning	[19]
*O. sativa*	*Pia*	Blast	*M. oryzae*	NBS-LRR	11	Map-based cloning	[72]
*O. sativa*	*Pi2*	Blast	*M. oryzae*	NBS-LRR	6	Map-based cloning	[70]
*O. minuta*	*Pi9*	Blast	*M. oryzae*	NBS-LRR	6	Map-based cloning	[70]
*O. sativa*	*Pi37*	Blast	*M. oryzae*	NBS-LRR	1	Map-based cloning	[69]
*O. rhizomatis*	*Pi54*	Blast	*M. oryzae*	CC-NBS-LRR	-	Map-based cloning	[73]
*O. sativa*	*Pib*	Blast	*M. oryzae*	NBS-LRR	2	Map-based cloning	[16]
*O. sativa*	*Pi-ta*	Blast	*M. oryzae*	NBS-LRR	12	Map-based cloning	[67]
*O. sativa*	*Pi-Kh*	Blast	*M. oryzae*	NBS-LRR	11	Map-based cloning	[68]
*O. sativa*	*Pid3*	Blast	*M. oryzae*	NBS-LRR	6	Map-based cloning	[71]

*O. minuta*—*Oryza minuta*, *O. rhizomatis*—*Oryza rhizomatis*, *O. sativa*—*Oryza sativa*, *M. oryzae*—*Magnaporthe oryzae*, *X. campestris*—*Xanthomonas campestris*, *X. oryzae*—*Xanthomonas oryzae* pv. *oryzae* (*Xoo*), CC—coiled-coil domain, NBS-LRR—nucleotide-binding site leucine-rich repeat, TIR—Toll/interleukin-1 receptor-like domain.

**Table 4:** Resistance genes, their donor parents, chromosomes location, and cloning techniques in *T. aestivum*.

Source	R-Gene	Disease	Pathogen	Gene Product	Chromosome	Cloning Technique	References
*T. aestivum*	*Pm1a*	Powdery mildew	*B. graminis*	CC-NBS-LRR	7AL	Map-based cloning, MutChromSeq	[103]
*T. aestivum*	*Pm2a*	Powdery mildew	*B. graminis*	CC-NBS-LRR	5DS	MutChromSeq	[21]
*T. aestivum*	*Pm2b*	Powdery mildew	*B. graminis*	CC-NBS-LRR	5DS	Map-based cloning	[100]
*T. aestivum*	*Pm3a* and *Pm3b*	Powdery mildew	*B. graminis*	CC-NBS-LRR	1AS	Map-based cloning	[93]
*T. aestivum*	*Pm3c* and *Pm3f*	Powdery mildew	*B. graminis*	CC-NBS-LRR	1AS	Map-based cloning	[94]
*T. aestivum*	*Pm4b*	Powdery mildew	*B. graminis*	Putative chimeric protein of a serine/threonine kinase and multiple C2 domains	2AL	MutChromSeq	[104]
*T. aestivum*	*Pm5e*	Powdery mildew	*B. graminis*	CC-NBS-LRR	7BL	Map-based cloning	[24]
*S. cereale*	*Pm8*	Powdery mildew	*B. graminis*	CC-NBS-LRR	1RS	Homology based cloning	[20]
*S. cereale*	*Pm17*	Powdery mildew	*B. graminis*	CC-NBS-LRR	1RS	Homology based cloning	[95]
*D. villosum*	*Pm21*	Powdery mildew	*B. graminis*	CC-NBS-LRR	6VS	Map-based cloning, MutRenSeq	[23]
*T. aestivum*	*Pm24*	Powdery mildew	*B. graminis*	A tandem kinase protein with putative kinase-pseudokinase domains	1DS	Map-based cloning	[96]
*T. turgidum* spp. *dicoccoides*	*Pm41*	Powdery mildew	*B. graminis*	CC-NBS-LRR	3BL	Map-based cloning	[97]
*T. urartu*	*Pm60a* and *Pm60b*	Powdery mildew	*B. graminis*	CC-NBS-LRR	7AL	Map-based cloning	[98]
*T. urartu*	*PmR1*	Powdery mildew	*B. graminis*	CC-NBS-LRR	7AL	Map-based cloning	[98]
*T. urartu*	*MlIW172*	Powdery mildew	*B. graminis*	CC-NBS-LRR	7AL	Map-based cloning	[91]
*T. aestivum*	*Pm38*/*Lr34*	Powdery mildew	*B. graminis*	ATP-binding cassette transporter	7DS	Map-based cloning	[79]
*T. aestivum*	*Pm46*/*Lr67*	Powdery mildew	*B. graminis*	Predicted hexose transporter	4DL	Map-based cloning	[78]
*T. aestivum*	*Lr10*	Leaf rust	*P. triticina*	CC-NBS-LRR	1A	Map-based cloning	[75]
*T. aestivum*	*Lr1*	Leaf rust	*P. triticina*	CC-NBS-LRR	5D	Map-based cloning	[77]
*A. tauschii*	*Lr21*	Leaf rust	*P. triticina*	CC-NBS-LRR	1D	Map-based cloning	[76]
*A. tauschii*	*Sr33*	Stem rust	*P. graminis*	CC-NBS-LRR	1D	Map-based cloning	[82]
*T. monococcum*	*Sr35*	Stem rust	*P. graminis*	CC-NBS-LRR	3A	Map-based cloning	[83]
*S. cereale*	*Sr50*	Stem rust	*P. graminis*	CC-NB-LRR	1RS	Map-based cloning	[84]
*T. turgidum* ssp. *durum*	*Sr13*	Stem rust	*P. graminis*	CC-NB-LRR	6AL	Map-based cloning	[86]
*T. monococcum*	*Sr21*	Stem rust	*P. graminis*	CC-NB-LRR	2A	Map-based cloning	[87]
*T. monococcum* ssp. *boeoticum*	*Sr22*	Stem rust	*P. graminis*	CC-NB-LRR	7AL	MutRenSeq	[85]
*A. tauschii*	*Sr45*	Stem rust	*P. graminis*	CC-NB-LRR	1DS	MutRenSeq	[85]
*A. tauschii* var*. meyeri*	*Sr46*	Stem rust	*P. graminis*	CC-NB-LRR	2DS	Map-based cloning	[81]
*T. monococcum*	*Sr60*	Stem rust	*P. graminis*	Wheat Tandem Kinase 2	5A	Map-based cloning	[88]
*T. aestivum*	*Cre3*	Cereal cyst	*H. avenae*	NBS-LRR	2D	Map-based cloning	[90]
*T. aestivum*	*Cre1*	Cereal cyst	*H. avenae*	NBS-LRR	2B	Map-based cloning	[90]
*T. aestivum*	*Yr10*	Stripe rust	*P. striiformis*	CC-NBS-LRR	1B	Map-based cloning	[102]
*T. aestivum*	*Yr36*	Stripe rust	*P. striiformis*	NBS-LRR	6B	Map-based cloning	[101]

*A. tauschii*—*Aegilops tauschii*, *A. thaliana*—*Arabidopsis thaliana*, *S. cereal*—*Secale cereal*, *T. aestivum*—*Triticum aestivum*, *T. monococcum*—*Triticum monococcum*, *T. turgidum*—*Triticum turgidum*, *T. urartu*—*Triticum Urart*, *B. graminis*—*Blumeria graminis*, *H. avenae*—*Heterodera avenae*, *P. graminis*—*Puccinia graminis*, *P. striiformis*—*Puccinia striiformis*, *P. triticina*—*Puccinia triticina*, CC—coiled-coil domain, NBS-LRR—nucleotide-binding site leucine-rich repeat, TIR—Toll/interleukin-1 receptor-like domain.

**Table 5:** Resistance genes, their donor parents, chromosomes location, and cloning techniques in *Z. mays*.

R-Gene	Disease	Pathogen	Gene Product	Chromosome	Cloning Technique	References
***Hm1***	Northern leaf spot	*C. carbonum*	NADPH HC toxin reductase	1	Transposon-induced mutagenesis	[15]
***Htn1***	Northern corn leaf blight	*S. turcica*	Receptor-like kinase	8	Map-based cloning	[105]
***Ht2***	Northern corn leaf blight	*S. turcica*	Receptor-like kinase	2	Map-based cloning	[106]
***Ht3***	Northern corn leaf blight	*S. turcica*	Receptor-like kinase	8	Map-based cloning	[106]
***Rp1-D21***	Southern corn rust	*P. polysora*	NBS-LRR	10	Transposon-induced mutagenesis	[107]
***RppC***	Southern corn rust	*P. polysora*	NBS-LRR	10	Map-based cloning	[108]
***ZmREM1.3***	Southern corn rust	*P. polysora*	Remorin protein		Map-based cloning	[109]
***RppK***	Southern corn rust	*P. polysora*	CC-NB-LRR	10	Map-based cloning	[110]
***RabGD1α***	Rough dwarf disease	*MRDD*	-	8	Map-based cloning	[111]
***ZmAuxRP1***	Gibberella stalk rot	*F. graminearum*	Stroma-localized auxin-regulated protein	1	Map-based cloning	[112]
***ZmCCT***	Gibberella stalk rot	*F. graminearum*	CCT-domain protein	10	Map-based cloning	[113]
***ZmFBL41***	Banded leaf and sheath blight	*R. solani*	F-box protein	4	Map-based cloning	[114]
***ZmMM1***	Northern leaf blightGray leaf spot Southern corn rust	*S. turcica* *C. zeae-maydis* *P. polysora*	MYB transcription factor	7	Map-based cloning	[115]
***ZmCCoAOMT2***	Gray leaf spot	*C. zeae-maydis*	Caffeoyl-CoA O-methyltransferase	9	Map-based cloning	[116]
***ZmWAK***	Head smut	*S. reilianum*	Receptor-like kinase		Map-based cloning	[117]
***ZmTrxh***	Mosaic	*SCMV*	h-type thioredoxin	3	Map-based cloning	[118]
***ZmABP1***	Mosaic	*SCMV*	Auxin-binding protein	3	Map-based cloning	[119]

MRDV—Maize rough dwarf disease, *SCMV*—*Sugarcane mosaic virus*, *C. zeae-maydis*—*Cercospora zeae-maydis*, *C. carbonum*—*Cochliobolus carbonum*, *F. graminearum*—*Fusarium graminearum*, *P. polysora*—*Puccinia polysora*, *R. solani*—*Rhizoctonia solani*, *S. turcica*—*Setosphaeria turcica*, *S. reilianum*—*Sporisorium reilianum*, CC—coiled-coil domain, NBS-LRR—nucleotide-binding site leucine-rich repeat.

**Table 6:** Resistance genes, their donor parents, chromosomes location, and cloning techniques in *Arabidopsis*.

R-Gene	Disease	Pathogen	Gene Product	Chromosome	Cloning Technique	References
*RPS2*	Downy mildew	*P. syringae*	CC-NBS-LRR	4	Map-based cloning	[39]
*RPM1*	Downy mildew	*P. syringae*	NBS-LRR	3	Map-based cloning	[40]
*RPP8/HRT*	Downy mildew	*P. parasitica*	NBS-LRR	5	Map-based cloning	[125]
*RPP13*	Downy mildew	*P. parasitica*	LZ NBS-LRR	3	Map-based cloning	[126]
*RCY1*	Mosaic	*CMV-Y*	CC-NBS-LRR	5	Map-based cloning	[136]
*RPP1*	Downy mildew	*P. parasitica*	TIR-NBS-LRR	3	Map-based cloning	[122]
*RPP4*	Downy mildew	*P. parasitica*	TIR-NBS-LRR	4	Map-based cloning	[124]
*RPS4*	Powdery mildew	*P. syringae*	TIR-NBS-LRR	5	Map-based cloning	[131]
*RPP5*	Downy mildew	*P. parasitica*	TIR-NBS-LRR	4	Map-based cloning	[123]
*RPS5*	Downy mildew	*P. parasitica*	NBS-LRR	1	Map-based cloning	[135]
*RRS1*	Bacterial wilt	*R. solanacearum*	TIR- NBS-LRR	5	Map-based cloning	[132]
*RFO1*	Fusarium wilt	*F. oxysporum*	Receptor-like kinase	1	Map-based cloning	[134]
*PBS1*	Powdery mildew	*P. syringae*	Serine/threonine kinase	5	Map-based cloning	[130]
*FLS2*	Powdery mildew	*P. syringae*	Receptor-like kinase	5	Map-based cloning	[120]
*BAK1*	Powdery mildew	*P. syringae*	Receptor-like kinase	4	Map-based cloning	[121]
*NDR1*	Powdery mildew/Downey mildew	*P. syringae*/*P. parasitica*	Plasma membrane-localized protein	3	Map-based cloning	[129]
*RPW8*	Powdery mildew	*E. cruciferarum*	NBS-LRR	3	Map-based cloning	[127]

*A. thaliana*—*Arabidopsis thaliana*, *E. cruciferarum*—*Erysiphe cruciferarum*, *F. oxysporum*—*Fusarium oxysporum*, *P. parasitica*—*Peronospora parasitica*, *P. syringae*—*Pseudomonas syringae*, *R. solanacearum*—*Ralstonia solanacearum*, *CMV*—*Cucumber mosaic virus*, CC—coiled-coil domain, NBS-LRR—nucleotide-binding site leucine-rich repeat, TIR—Toll/interleukin-1 receptor-like domain.

**Table 7:** Resistance genes, their donor parents, chromosomes location, and cloning techniques in *S. lycopersicum*.

Source	R-Gene	Disease	Pathogen	Gene Product	Chromosome	Cloning Technique	References
*S. lycopersicum*	*Pto*	Bacterial speck	*P. syringae*	Serine-threonine kinase	5	Map-based cloning	[38]
*S. pimpinellifolium*	*Prf*	Bacterial speck	*P. syringae*	LZ-NBS-LRR	5	Map-based cloning	[140]
*S. peruvianum*	*Mi*	Root knot	*M. javanica*	NBS-LRR	6	Map-based cloning	[143]
*S. lycopersicum*	*I2*	Fusarium wilt	*F. oxysporum*	LZ-NBS-LRR	11	Map-based cloning	[142]
*S. pimpinellifolium*	*Ph-1*, *2* and *3*	Late blight	*P. infestans*	CC-NBS-LRR	9	Map-based cloning	[147]
*S. peruvianum*	*Sw-5*	Tomato spotted wilt	*TSWV*	NBS-LRR	9	Map-based cloning	[141]
*S. lycopersicum*	*Tm-2*	Tobacco mosaic	*TMV*	NBS-LRR	9	transposon tagging	[145]
*S. lycopersicum*	*Bs4*	Bacterial spot	*X. campestris*	TIR-NBS-LRR	5	Map-based cloning	[146]
*S. pimpinellifolium*	*Hero*	Potato cyst	*G. rostochiensis*	NBS-LRR	4	Map-based cloning	[144]
*S. pimpinellifolium*	*Cf-2*	Leaf mold	*C. fulvum*	NBS-LRR	6	Map-based cloning	[11]
*S. peruvianum*	*Cf-4*	Leaf mold	*C. fulvum*	NBS-LRR	1	Map-based cloning	[12]
*S. peruvianum*	*Cf-5*	Leaf mold	*C. fulvum*	NBS-LRR	6	Map-based cloning	[139]
*S. pimpinellifolium*	*Cf-9*	Leaf mold	*C. fulvum*	NBS-LRR	1	Transposon tagging (Ac-Ds system)	[10]
*S. lycopersicum*	*Ve1,2*	Verticillium wilt	*V. dahliae*	Receptor-like kinase	9	Map-based cloning	[14]
*S. lycopersicum*	*Hcr9-4E*	Leaf mold	*C. fulvum*	Receptor-like kinase	1	Map-based cloning	[12]
*S. pimpinellifolium*	*Fen*	Bacterial speck	*P. syringae*	Serine/threonine kinase	5	Map-based cloning	[138]
*S. lycopersicum*	*Pti1*	Bacterial speck	*P. syringae*	Serine/threonine kinase	12	Map-based cloning	[137]

*S. lycopersicum*—*Solanum lycopersicum*, *S. pimpinellifolium*—*Solanum pimpinellifolium*, *S. peruvianum*—*Solanum peruvianum*, *C. fulvum*—*Cladosporium fulvum*, *F. oxysporum*—*Fusarium oxysporum*, *G. rostochiensis*—*Globodera rostochiensis*, *M. javanica*—*Meloidogyne javanica*, *P. syringae*—*Pseudomonas syringae*, *V. dahliae*—*Verticillium dahlia*, *TMV*—*Tobacco mosaic virus*, *TSWV*—*Tomato spotted wilt virus*, Ac-Ds system—Activator and Dissociator system of Maize, CC—coiled-coil domain, NBS-LRR—nucleotide-binding site leucine-rich repeat, TIR—Toll/interleukin-1 receptor-like domain.

**Table 8:** Commonly used sgRNA design tools and databases in plant genome editing.

Name	Cas Nuclease Enzyme	Major Features	Website
CRISPOR	Cas9 orthologues and Cas variants	Cloning, expressing, and validating sgRNA sequences for the CRISPR/Cas9 system, as well as providing primers needed for testing guide activity and target validation	http://crispor.tefor.net/ (accessed on 3 May 2023)
CHOPCHOP	Cas9, Cas12, Cpf1, and TALEN	It provides multi-targeting systems, such as knockout, knock-in, gene activation, and repression. It allows for the design of sgRNAs in a specific region, 5′ UTR, 3′ UTR, promoter, or the gene coding region	https://chopchop.cbu.uib.no/ (accessed on 3 May 2023)
CRISPR RGEN Tools	Cas9 orthologues and Cas variants	It provides multiple sgRNA design tools with high accuracy	http://www.rgenome.net/cas-designer/ (accessed on 3 May 2023)
E-CRISP	SpCas9	It targets any nucleotide sequence of the genome. It also checks for target specificity of the putative designs and their genomic context (e.g., exons, transcripts, CpG islands)	http://www.e-crisp.org/E-CRISP/index.html (accessed on 3 May 2023)
CRISPR-GE	SpCas9, FnCpf1, and AsCpf1	It predicts the specificity of a target site and the design sgRNAs for different CRISPR/Cas systems. It also provides a primer design tool for vector construction and mutant detection	http://skl.scau.edu.cn/ (accessed on 3 May 2023)
CRISPR-P	Cas9 and variants	It provides on-target and off-target scoring and gRNA sequence analysis. It allows one to choose U3 or U6 sgRNA promoter-driven expression cassettes for designing sgRNA	http://crispr.hzau.edu.cn/CRISPR2/ (accessed on 3 May 2023)
CRISPR-PLANT V2	SpCas9	It allows for the design and construction of sgRNAs for CRISPR-Cas9-mediated genome editing	https://www.genome.arizona.edu/crispr2/ (accessed on 3 May 2023)
CRISPRlnc	SpCas9	It provides a downloadable validated sgRNA database	http://www.crisprlnc.org/ (accessed on 3 May 2023)
SNP-CRISPR	NGG, NAG, and PAM	It allows for the design of sgRNAs for targeting SNPs or Indels	https://www.flyrnai.org/tools/snp_crispr/web/ (accessed on 3 May 2023)

## Data Availability

Data sharing not applicable.
